# Saucerization Combined With Meniscal Repair Is an Effective Surgical Technique for the Treatment of Symptomatic Bilateral Medial Discoid Meniscus With Good Short‐ to Medium‐Term Results: A Case Report

**DOI:** 10.1155/cro/1851165

**Published:** 2025-11-28

**Authors:** Juan José Martínez-Arboleda, Juanita Villalba-Reyes, Alejandro Delgado-Cortez, Giusseppe Aguado-Gómez, Juan David Parra-Hernandez

**Affiliations:** ^1^ Orthopaedics and Traumatology Residency Program, Faculty of Health Sciences, Pontificia Universidad Javeriana Cali, Cali, Colombia; ^2^ Medicine Program, Faculty of Medicine, Universidad de La Sabana, Bogota, Colombia, unisabana.edu.co; ^3^ Shoulder and Knee Arthroscopic Surgery Program, Faculty of Health Sciences, Pontificia Universidad Javeriana Cali, Cali, Colombia; ^4^ Department of Orthopaedics and Traumatology, Clínica Imbanaco, Cali, Colombia

**Keywords:** arthroscopy, knee, Lysholm knee score, meniscus, rare diseases

## Abstract

**Background:**

The discoid meniscus is an anomaly caused by a congenital morphological variant of the meniscus, in which the meniscus has an abnormally wide and flat cartilage shape. An arthroscopic partial meniscectomy (saucerization) is the preferred treatment strategy to maintain peripheral stability and functionality of the meniscus. Meniscal repair is indicated as an additional surgical strategy when residual instability occurs after saucerization and in some rupture patterns, such as horizontal tears.

**Case Report:**

We present the first case reported in Colombia (Latin America) of a 19‐year‐old patient with a diagnosis of a bilateral medial discoid meniscus with instability and a horizontal tear in the body, anterior and posterior horns who was managed with meniscal saucerization and repair (each knee at different times) satisfactorily with good 1‐year follow‐up clinical and functional results.

**Conclusions:**

Saucerization combined with meniscal repair is an effective surgical technique for treating a symptomatic bilateral medial discoid meniscus with a horizontal tear and instability with good short‐ to medium‐term results.

## 1. Introduction

Discoid meniscus is an anomaly caused by a congenital morphological variant of the meniscus, which has an abnormally wide and flat cartilaginous shape. It can occur in any of the menisci of the knee. However, it is less frequent in the medial (0.06%–3%) than in the lateral (1.2%–5.2%) [1, 2] and even less frequent bilaterally (0.012%) [3].

Since Murdoch reported the first case of bilateral medial discoid meniscus in 1956, there have been only 24 cases reported in the literature [3]. Although different management techniques have been proposed, arthroscopic partial meniscectomy (saucerization) is preferred to maintain peripheral stability and meniscal function [4, 5]. Meniscal repair is indicated as an additional surgical strategy when residual instability persists after saucerization and in some rupture patterns, such as horizontal tears [6].

This article reports the case of a young patient with a diagnosis of unstable bilateral medial discoid meniscus managed with arthroscopic partial meniscectomy (saucerization) and meniscal repair with satisfactory short to medium‐term results, this being the first case reported in Colombia and one of the few in Latin America [7].

## 2. Case Presentation

A previously healthy, 19‐year‐old male patient presented to the office due to 5‐year bilateral knee pain (predominantly on the right) of insidious onset with no apparent trigger. He played soccer but was frequently in discomfort during this activity and had used non‐steroidal anti‐inflammatory drugs to facilitate participation in this sport. Due to partial improvement of pain due to medication, he had not had previous assessments by a physician. Physical examination of both knees showed adequate alignment, ranges of mobility of 0°–140°, no joint effusion, negative anterior and posterior drawer tests and negative lateral instability. However, he presented pain in the medial joint line and a positive McMurray′s test for a bilateral medial meniscus. The Lysholm score (validated in our country’s language) [8] was used to evaluate the patient’s functionality. The right knee presented a Lysholm score of 52, and the left knee was 57 (Table 1).

**Table 1:** Lysholm score before and after surgery (1 year follow‐up).

	**Pre-surgical**	**1 year follow-up**
Right knee Lysholm score ^∗^	52	92
Left knee Lysholm score ^∗^	57	94

^∗^Scores ranging from 0 to 100: 95–100 Excellent, 84–94 Good, 65–83 Fair, <65 Poor.

Bilateral weight‐bearing radiographs of the knees had no significant findings. Magnetic resonance imaging of the right knee evidenced a horizontal tear in the body, anterior and posterior horn of the medial meniscus, and a partial dislocation of the medial meniscus towards the medial intercondylar eminence **(**Figure 1 **)**. According to the Watanabe classification [9] (which divides the discoid menisci into complete, incomplete and Wrisberg ‐ligament variants), this was a complete discoid meniscus. The findings in the left knee were similar.

Figure 1Coronal and sagittal (a) magnetic resonance imaging of the right knee showing a medial discoid meniscus with a horizontal rupture in the body, involvement of the anterior and posterior horns, and partial dislocation of the medial meniscus toward the medial intercondylar eminence. Bilateral weight‐bearing radiographs of the knees (b) show no significant findings.

**Figure:**
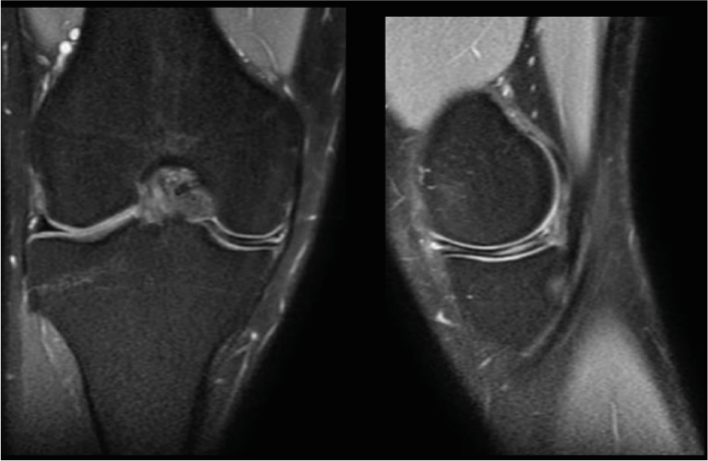
(a)

**Figure:**
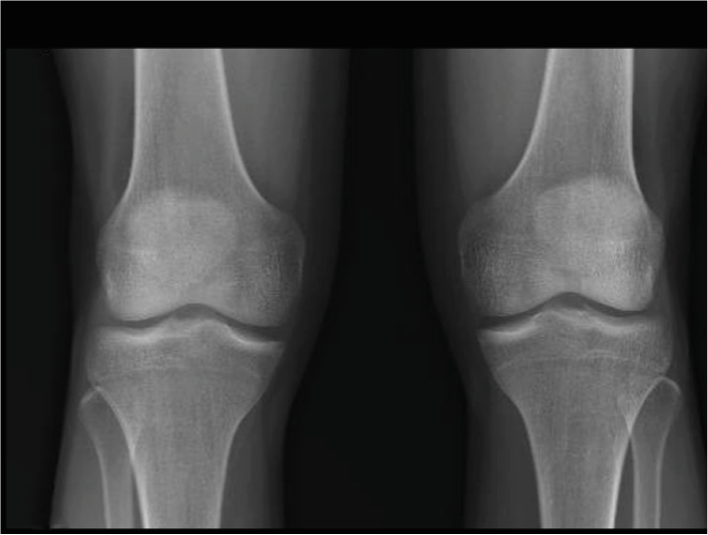
(b)

Considering the clinical and imaging findings, a diagnosis of unstable bilateral medial discoid meniscus was established. The patient was initially managed conservatively under the supervision of a sports physiotherapist. The rehabilitation programme consisted of physiotherapy sessions three times per week, focusing on progressive quadriceps and hamstring strengthening, proprioceptive balance training and restoration of knee range of motion. After 3 months, the patient was instructed to continue with a home‐based exercise regimen on alternate days, which included closed kinetic chain exercises. Temporary restriction from running, pivoting and contact sports was advised, alongside short courses of nonsteroidal anti‐inflammatory medication for symptomatic relief. Despite adherence to this programme for 11 months, the patient continued to experience pain and mechanical discomfort. As a result, the treating physician proposed arthroscopic partial meniscectomy (saucerization) with peripheral repair of the medial meniscus of the right knee, which was the more symptomatic side. The surgical plan was agreed upon through a shared decision‐making process between the surgeon and the patient, and the procedure was performed following informed consent.

## 3. Surgical Technique

**Figure 2:**
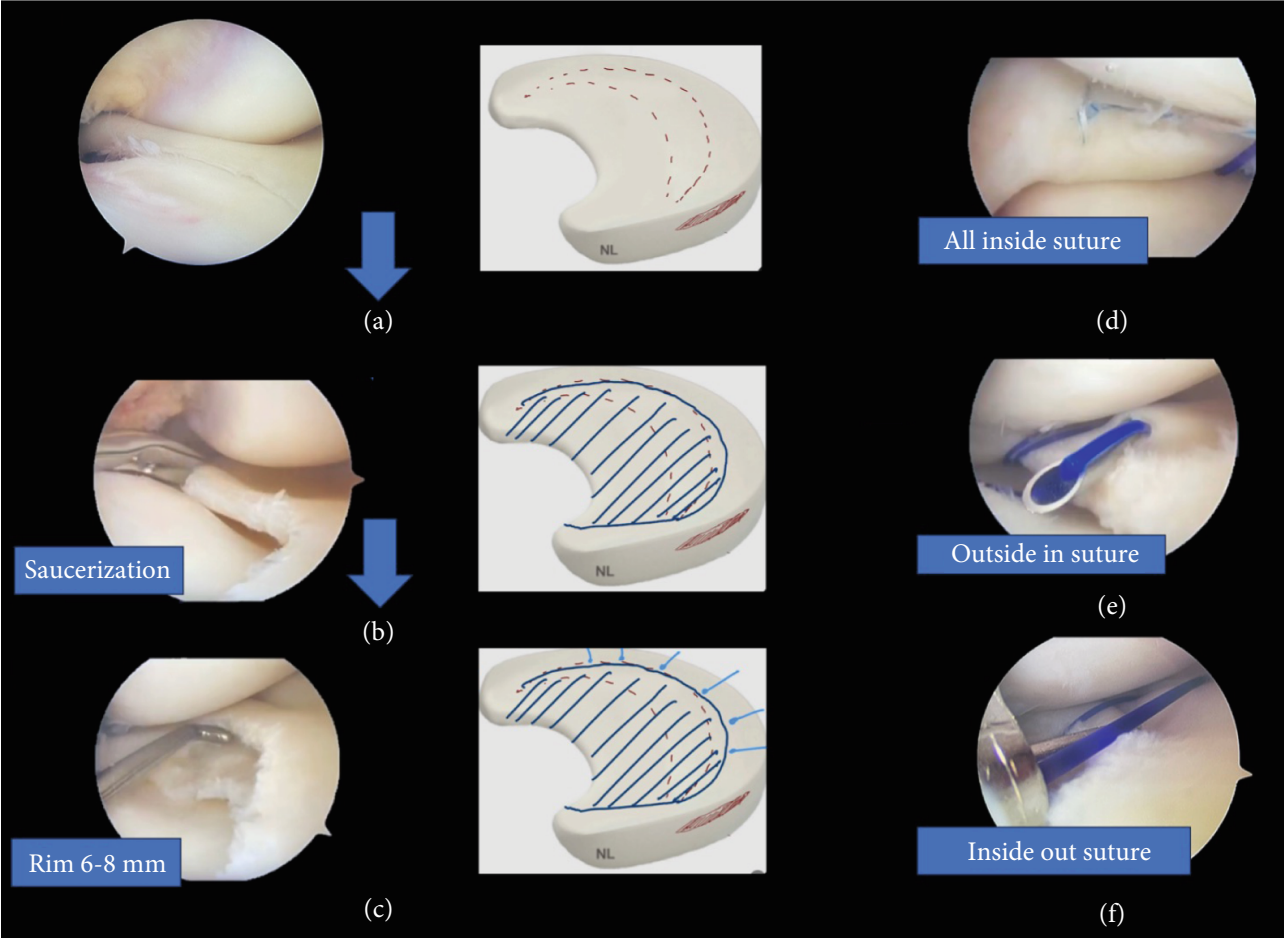
Arthroscopic images of the right knee. The medial discoid meniscus is subluxed toward the intercondylar notch (a). Saucerization of the body, anterior, and posterior horns (b). Unstable 6–8 mm rim after saucerization (c). All‐inside suture of the posterior horn (d). Outside‐in suture of the anterior horn (e). Inside‐out suture of the body (f).

**Figure 3:**
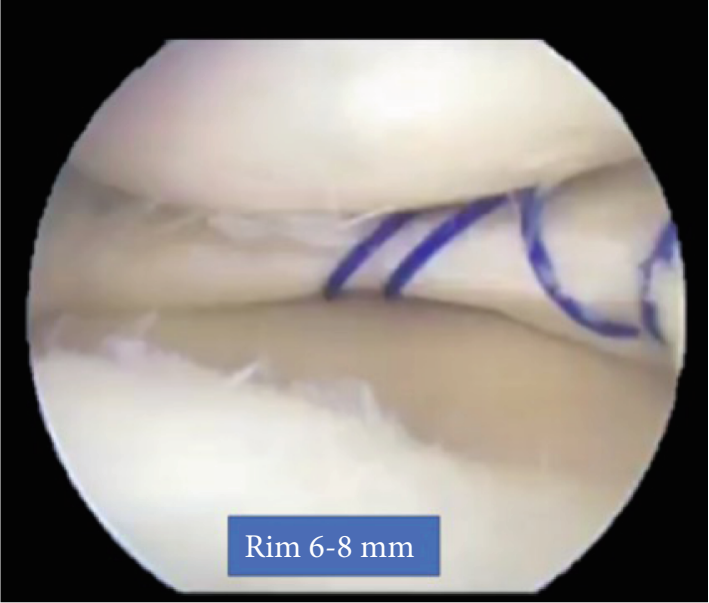
Arthroscopic images of the right knee. Medial discoid meniscus after saucerization and repair.

Under general anesthesia, a right knee arthroscopy using standard anterolateral and anteromedial portals was performed. A medial discoid meniscus with a horizontal tear in the body, anterior and posterior horn was found. We proceeded to perform a meniscal saucerization of the body and posterior horn preserving a meniscal rim of 6–8 mm **(**Figures 2 and 3 **)**. Due to residual instability following saucerization, meniscal repair was indicated. An all‐inside suture with two longitudinal sutures at the level of the posterior horn (Fast‐Fix 360 system, Smith & Nephew) was performed. Subsequently an inside‐out (with three longitudinal sutures) at the level of the body and an outside‐in (with three longitudinal sutures) sutures at the level of the anterior horn were made **(**Figure 2 **)**. After stability confirmation was verified with a probe, the surgical portals were closed.

## 4. Rehabilitation Protocol

During the first 4 weeks, the patient was allowed to ambulate with crutches with restricted weight bearing with a mobility of 0°–90°. Between the fourth and sixth weeks, partial support with crutches and mobilities of 90°–120° were allowed. From the sixth week, the patient had complete support of the limb and a full range of knee movement. From the third month, the patient started strengthening exercises, and after 6 months, impact sports and pivot activities were allowed.

## 5. Follow‐Up and Results

The postoperative course was favourable, with no postoperative problems observed. Then, 6 weeks postoperatively, the patient walked with no crutches, a knee flexion of 140**°**, no extension deficit and minimal quadriceps hypotrophy. At 1 year of follow‐up, the patient was almost asymptomatic (right knee) and could perform his sports activity (soccer) with moderate limitation derived from his left knee. The same surgical procedure was performed in the left knee 4 months later, with no complications and comparable clinical progress. The patient could resume his sports activity at the same level as before the onset of symptoms after 1 year postoperatively of the left knee. The Lysholm score at 1‐year follow‐up for the right knee was 92, and for the left knee, 94 (Table 1 **).**

### 5.1. Ethical Considerations

This research was carried out following the ethical principles established in the Helsinki Declaration. Informed consent was obtained from the patient authorizing this case report.

## 6. Discussion

It is important to present this case of complete bilateral medial discoid meniscus with intrasubstance horizontal degeneration as it is a very uncommon cause of knee pain and functional limitation in the general population. This is the first case reported in Colombia and one of the few reported in Latin America. The patient experienced a satisfactory clinical recovery following saucerization and repair of the medial meniscus in both knees.

The clinical presentation of a medial discoid meniscus is similar to that of a lateral discoid meniscus, where there is pain and mechanical symptoms such as locking [6, 10]. In our case, pain was predominant, and although the torn meniscus was subluxed towards the intercondylar groove, the patient did not report mechanical blockage or limitation of flexion and extension. Our patient also presented a horizontal meniscus tear in all segments, the most common associated tear pattern reported in the literature [6, 10].

Regarding management, meniscectomy has been, for many years, the standard treatment for symptomatic discoid meniscus. However, although several studies have demonstrated that total meniscectomy is associated with a higher risk of osteoarthritis compared with partial meniscectomy in anatomically normal menisci [5, 11], these findings may not be directly applicable to discoid variants due to their distinct morphology and altered load distribution. Nevertheless, the current trend in meniscal surgery favored tissue preservation whenever possible, with partial meniscectomy (saucerization) and repair increasingly employed to maintain peripheral stability and long‐term joint function.

Arthroscopic saucerization and peripheral suture repair are additional surgical interventions recommended for painful discoid meniscus [12, 13]. Some authors recommended preserving the discoid meniscus in asymptomatic or minimally symptomatic patients and encouraged saucerization of a torn symptomatic discoid meniscus [14]. Flouzat‐Lachaniette et al. [2] described four cases of discoid medial menisci treated with meniscoplasty, removing only the central part of the meniscus, and one case with associated meniscal repair demonstrating satisfactory results. Cho [15] reported a case of bilateral discoid medial menisci with unilateral symptoms.

Likewise, in our instance, we executed saucerization of the hypertrophic regions and repaired the symptomatic discoid medial meniscus‐related lesions. The central portion of the discoid meniscus was saucerized until a consistent semilunar shape with a 6–8 mm width was obtained. Similar to Cho [15], we also repaired the anterior horn, which exhibited instability and a horizontal rupture but with an outside‐in suture.

Short‐term outcomes following isolated simple saucerization have generally been favorable in the literature. For example, Feroe et al., in a case series of 12 knees (8 patients) with medial discoid meniscus, performed only saucerization to manage four knees [6]. Only one knee that underwent simple saucerization developed arthrofibrosis and required a subsequent operation for lysis of adhesions. These patients were cleared for athletic participation at a median of 4 months following their index operation, and more than half of the total cases achieved complete resolution of symptoms by their final follow‐up at a mean of 19 months out from surgery. Our patient also underwent meniscal repair, and his clinical outcome was good; he was practically asymptomatic 1 year after surgery, had an increase of more than 20 points in the Lysholm score of both knees and could return to his sporting activity.

Meniscal repair (in the context of a non‐discoid meniscus) has achieved consensus in recent decades. Pursuing meniscal repair for all reparable tears, particularly in active and younger adults, is advised [16]. Arthroscopic repair should be indicated especially for horizontal meniscal and root tears due to its favourable clinical outcomes [17, 18]. Ogawa et al. showed that, despite the suboptimal healing of horizontal meniscus tears, arthroscopic repair of these lesions should be pursued, with favourable clinical outcomes [17]. The 2019 consensus on the management of traumatic meniscus injuries indicated that many meniscus tears previously deemed irreparable should be repaired [17].

A similar approach has been applied in managing the medial discoid meniscus, especially in meniscal instability and horizontal tears, to stabilize the meniscal remnant after saucerization [6, 10]. Outcomes following saucerization requiring concomitant meniscal repair are less favorable than in a lesion of a non‐discoid meniscus. In the case series of Feroe et al., seven medial menisci were managed with saucerization and meniscal repair. Four of the seven cases required a subsequent revision. Only these patients (in addition to the other one that resulted in arthrofibrosis) had symptoms at the last clinical follow‐up. However, the results of Feroe et al. should be interpreted with caution. As previously mentioned, the indication to perform a repair concomitant to saucerization is a horizontal tear and, in this case, also residual instability, so these patients have more severe lesions than those who only undergo saucerization. Therefore, the frequency of specific outcomes, such as re‐intervention of these two types of management, is not entirely comparable since the patients who undergo one or the other kind of management present the same pathology but with different severity.

Like Feroe et al. [6], we agree that patients should be counselled to define the postoperative expectations of possible reintervention if saucerization and repair are decided as management. Not because of the procedure itself, but because if it is performed, it is because there is residual instability and a horizontal tear, indicative of a more severe pathology. In our case, the treating physician thoroughly explained the procedure to the patient, and the surgical strategy resulted from a shared decision‐making process.

## 7. Conclusions

Saucerization combined with meniscal repair is a promising therapeutic strategy for the treatment of medial discoid meniscus with a horizontal tear and residual instability after saucerization, and has been shown to yield good short‐ to medium‐term results. The decision of this therapeutic strategy should be the result of a shared decision‐making process between the surgeon and the patient.

## Consent

The authors attest that consent has been obtained from any patient(s) appearing in this publication. If the individual may be identifiable, the authors have included a statement of release or other written form of approval from the patient(s) with this submission for publication.

## Conflicts of Interest

The authors declare no conflicts of interest.

## Funding

No funding was received for this manuscript.

## Data Availability

The data that support the findings of this study are available on request from the corresponding author. The data are not publicly available due to privacy or ethical restrictions.
